# An Enforced Anti-Spitting Law

**Published:** 1899-10

**Authors:** 


### An Enforced Anti-Spitting Law.

The anti-expectoration ordinance in San Francisco seems likely to hold its own and to be enforced without fear or favor. Ohe offender, who is reported to be a millionaire, has had to undergo his punishment, and the repetition of the offense only made it more severe. If this is not merely an indication of a "sand lots" prejudice against millionaires, and if like even justice is dealt out to the hoodlum who deserves it, as well as to every grade between, San Francisco is to be congratulated. The prohibition of indiscriminate expectoration is one of the most reasonable hygienic regulations, and has, moreover, the advantage of being cosmetic as well as sanitary. It is to be hoped that this pervasive nuisance can be legally checked elsewhere as well as in San Francisco.—*The Journal of the American Medical Association.*

Absolute dryness prevents the development of germ life.

It is difficult to freeze a germ to death, but boiling quickly destroys all micro-organisms.

